# Synthesis and Characterization of MWCNT-COOH/Fe_3_O_4_ and CNT-COOH/Fe_3_O_4_/NiO Nanocomposites: Assessment of Adsorption and Photocatalytic Performance

**DOI:** 10.3390/nano12173008

**Published:** 2022-08-30

**Authors:** Adina Stegarescu, Humberto Cabrera, Hanna Budasheva, Maria-Loredana Soran, Ildiko Lung, Francesca Limosani, Dorota Korte, Matteo Amati, Gheorghe Borodi, Irina Kacso, Ocsana Opriş, Monica Dan, Stefano Bellucci

**Affiliations:** 1 National Institute for Research and Development of Isotopic and Molecular Technologies, 67-103 Donat, 400293 Cluj-Napoca, Romania; 2 Optics Lab, STI Unit, The Abdus Salam International Centre for Theoretical Physics, Costiera 11, 34151 Trieste, Italy; 3 Laboratory for Environmental and Life Sciences, University of Nova Gorica, Vipavska 13, SI-5000 Nova Gorica, Slovenia; 4 INFN-National Laboratories of Frascati, Via Enrico Fermi 54, 00044 Frascati, Italy; 5 Department of Information Engineering, Polytechnic University of Marche, Via Brecce Bianche, 1, 60131 Ancona, Italy; 6 Spectroscopy, Photoemission and Dynamics, Elettra—Sincrotrone Trieste S.C.p.A. S.S. 14, km 163.5 in Area Science Park, Basovizza, 34149 Trieste, Italy

**Keywords:** carbon nanotubes, magnetite, tartrazine, photodegradation, adsorption, water decontamination

## Abstract

In this study the adsorption and photodegradation capabilities of modified multi-walled carbon nanotubes (MWCNTs), using tartrazine as a model pollutant, is demonstrated. MWCNT-COOH/Fe_3_O_4_ and MWCNT-COOH/Fe_3_O_4_/NiO nanocomposites were prepared by precipitation of metal oxides in the presence of MWCNTs. Their properties were examined by X-ray diffraction in powder (XRD), Fourier-transform infrared spectroscopy (FT-IR), transmission electron microscopy (TEM), scanning electron microscopy (SEM), Raman spectroscopy, synchrotron-based Scanning PhotoElectron Microscopy (SPEM), and Brunauer-Emmett-Teller (BET) analysis. It was found that the optimal adsorption conditions were pH 4 for MWCNT-COOH/Fe_3_O_4_ and pH 3 for MWCNT-COOH/Fe_3_O_4_/NiO, temperature 25 °C, adsorbent dose 1 g L^−1^, initial concentration of tartrazine 5 mg L^−1^ for MWCNT-COOH/Fe_3_O_4_ and 10 mg L^−1^ for MWCNT-COOH/Fe_3_O_4_/NiO and contact time 5 min for MWCNT-COOH/Fe_3_O_4_/NiO and 15 min for MWCNT-COOH/Fe_3_O_4_. Moreover, the predominant degradation process was elucidated simultaneously, with and without simulated sunlight irradiation, using thermal lens spectrometry (TLS) and UV–Vis absorption spectrophotometry. The results indicated the prevalence of the photodegradation mechanism over adsorption from the beginning of the degradation process.

## 1. Introduction

Multi-walled carbon nanotubes (MWCNTs) were discovered by Iijima in 1991 [1] and became a very promising material in the field of agronomy, medicine, electronics and depollution processes. Thus, they are intensively studied from the scientific community, alone or in combination with other nanomaterials [2]. MWCNT is a highly porous material and has a hollow structure, high surface area, thermal stability, good thermal and electrical conductivity and optical activity, as well as mechanical damage resistance. It also has a strong ability to establish interactions with dyes or other organic materials. The continuous decrease in its price makes it an important nanomaterial for water and environmental decontaminations [3,4,5,6,7,8]. The properties of MWCNT can be further improved by fabricating a complex with Fe_3_O_4_; thus, combining its mechanical and electrical properties, as well as its thermo-optical properties with the superparamagnetic behavior of Fe_3_O_4_. In such a way, a high-strength magnetic material can be produced. It can then be applied in medicine, electrical devices, and magnetic data storage systems and in heterogeneous catalysis. Furthermore, in the case of environmental decontamination, such a material can become an alternative to other materials (e.g., silica, polyurethane foam, cellulose, biological substances) [2,9,10,11,12].

Luo et al. in 2016 [13] found that using transition metal oxides (MnO_2_, CuO, Co_3_O_4_, NiO) and their hybrids in combination with carbon nanotubes (CNTs), it was possible to improve the electrochemical properties of the final materials with good and promising results in the field of electrode materials for a lithium-ion battery. Rao et al. in 2017 [14] presented the efficiency of CuO nanoparticles in photocatalytic activity for tartrazine degradation under visible light illumination, demonstrated by GC/MS analysis. Functionalization of CNT membranes with other antimicrobial nanoparticles, such as silver nanoparticles, CuO, MnO_2_ and TiO_2_, is a promising route for water and environmental depollution because it increases the self-cleaning capacities of the final material [15,16]. This is of high importance, since intensive utilization of dyes by different industries in various processes is a major source of environmental pollution, due to their resistance to light degradation. Thus, it is difficult to degrade these dyes from wastewater. Among all the pollutants, tartrazine is one of the most commonly used in a variety of food products as a coloring agent. Thus, it is very important to determine and remove it from food products and waters [17]. The scientific community has developed different decontamination protocols (e.g., adsorption, coagulation, flocculation, ozonation, photocatalysis) to reduce negative impacts on the environment. Adsorption and photodegradation are the most intensively studied, since they are easy to perform [18,19,20].

Soylaka and Cihan in 2013 [17] used MWCNT for recovery of tartrazine for the first time. They used spectrophotometric determination of the amount of tartrazine in network drinking water, fruit juice, and powdered beverages. They found that 200 mg of MWCNT (as a solid-phase) can be used at least 200 times for successful removal of tartrazine. Thus, it is a green and simple method, comparable to methods used so far for such purposes [17,21,22,23,24].

Nait-Merzouga et al. in 2017 [25] examined the adsorption capacity of tartrazine by commercial activated carbon and an oxygen functionalized MWCNT (O-MWCNT). They modified different parameters of the adsorption process as the contact time, initial concentration of tartrazine, amount of adsorbent, pH and temperature. They found that O-MWCNT worked better in the process of tartrazine adsorption, due to its higher specific surface area than commercial activated carbon [25].

Khan et al. in 2018 [26] prepared nanocomposites of MWCNTs loaded with ZnO/NiO by means of a co-precipitation method, that were successfully used as photocatalysts in the process of textile azo dye degradation. The photodegradation ability of MWCNTs loaded with ZnO was higher than a single ZnO material. The addition of a small quantity of NiO (3%) onto the surface of MWCNT-ZnO, increased the photocatalytic activity of the whole composite, but further study is needed to determine the exact role of NiO in the photodegradation process [26].

Other promising materials for dye degradation are TiO_2_ in the form of nanoparticles, and TiO_2_ modified using metallic elements, such as Cu and Zr, or in combination with MWCNT [27,28]. The binary system MWCNT/TiO_2_ was found to be the most efficient material for photodegradation of colored textile wastewater dyes. Due to the synergic effect of MWCNT/TiO_2_ nanoparticles applied as photocatalysts, the use of high pressure of oxygen or heating was not required [27]. MWCNT/TiO_2_ nanocomposites, under visible light, have the ability to decolor different types of dyes (e.g., azo dye, methylene blue, methyl orange or other dyes) in aqueous solutions, with much higher efficiency than the mechanical mixture of the single nanoparticles (MWCNT and TiO_2_) [29,30,31,32]. It was also found that MWCNT/ZnO nanocomposite demonstrated photocatalytic activity against some dyes (e.g., rhodamine B, azo-dyes, methylene blue or methylene orange), as well as against other classes of pollutants (acetaldehyde and cyanide). The material photocatalytic properties are associated with the type of synthesis, due to the difference of surface states resulting from the different conditions of its preparation [31,33,34]. It was concluded that UV, visible light and sunlight photocatalytic activity of the nanocomposites based on MWCNT/metal oxide were higher than that of the individual components [29,30,32]. Although both absorption and photodegradation capabilities of MWCNT/metal oxide have been demonstrated separately, there is a lack of detailed investigation on the simultaneous performance of these mechanisms in the degradation efficiency of dye pollutants. The novelty of this study consists of assessment of simultaneous removal of tartrazine by absorption and photodegradation. Also, the MWCNT-COOH/Fe_3_O_4_/NiO nanocomposite is new.

The aim of this study was focused on the synthesis, characterization and application of magnetic nanocomposites, MWCNT-COOH/Fe_3_O_4_ and MWCNT-COOH/Fe_3_O_4_/NiO, as adsorbents and photocatalysts, for removal of tartrazine from aqueous solutions. In order to establish the conditions for the optimal retention of tartrazine, the influence of some physico-chemical parameters on the adsorption process were evaluated. Among the examined parameters were the following: the initial pH, temperature, adsorbent dose, and contact time, as well as the initial concentration of the dye solution. Furthermore, analysis of the tartrazine photodegradation process was also performed. We compared the experimental results to elucidate which mechanism, adsorption or photodegradation, dominates the degradation process.

## 2. Materials and Methods

### 2.1. Materials

In the synthesis of nanocomposites, MWCNT (D × L 110–170 nm × 5–9 μm), NiCl_2_ × 6H_2_O, ascorbic acid, cetyltrimethylammonium bromide (CTAB) were purchased from Sigma-Aldrich (Schnelldorf, Germany), FeCl_3_ × 6H_2_O from Alfa Aesar (Kandel, Germany), FeSO_4_ × 7H_2_O from VWR Chemicals (Wien, Austria) and NH_3_ 25% solution from Chemical Company (Iaşi, Romania). Tartrazine was chosen as pollutant in this study and was purchased from Sigma-Aldrich (Schnelldorf, Germany). The pH adjustment was performed with HCl and NaOH that were purchased from Sigma-Aldrich (Schnelldorf, Germany) and VWR Chemicals (Wien, Austria), respectively. Aqueous solutions were prepared using ultrapure water (Direct-Q^®^ 3 UV Water Purification System, Merck, Darmstadt, Germany).

### 2.2. Synthesis of Nanocomposites

#### 2.2.1. MWCNT-COOH/Fe_3_O_4_ Synthesis

MWCNT functionalized with COOH groups [35] were stirred in an ultrasonic bath for 20 min in water in a ratio of 1.66:1 (*w/v*). After that time, the stirring process was continued on a magnetic plate, under argon, for 30 min at 60 °C. An amount of 0.34 moles of FeCl_3_ × 6H_2_O was added to the obtained suspension and the stirring was continued for another 30 min, then 0.17 moles FeSO_4_ × 7H_2_O was added to the solution and stirred for a further 30 min. In the end, 18 mL of 6% NH_4_OH were added to the mixture in rare droplets and stirred for another 2 h. The prepared nanocomposite was washed by centrifugation with water until the pH was neutral, and then dried overnight in an oven at 60 °C.

#### 2.2.2. MWCNT-COOH/Fe_3_O_4_/NiO Synthesis

The mixture of CNT-COOH/Fe_3_O_4_ was sonicated for 30 min in water, in a ratio of 1.66:1 (*w/v*). After that, freshly prepared solutions of 0.1249 g NiCl_2_ × 6H_2_O in 50 mL of water and 0.9687 g of ascorbic acid in 50 mL of water were added. After the addition of 0.5467 g of CTAB to the previously obtained suspension, another 50 mL of water was added and the whole mixture had its pH adjusted to the value of 6.5 by NaOH solution. Then, it was heated to 85 °C and further stirred for another 3 h. Finally, the sample was washed with water by centrifugation and dried in an oven at 75 °C.

### 2.3. Nanocomposite Characterization

The structural characterization of the synthesized nanocomposites was performed using a D 8 Advance diffractometer in Bragg Brentano geometry. An X-Ray Cu tube with a Ge (111) monochromator was used in the incident beam to obtain only Cu Kα1 radiation and a LynxEye type position detector. The scan was performed at an angle of 20–850 (2θ) with a step of 0.020, and with a time per step of 1s.

Surface chemical information at the micron and submicron scales were obtained via synchrotron-based Scanning PhotoElectron Microscopy (SPEM), at the ESCA Microscopy beamline at Elettra synchrotron facility (Trieste, Italy) [36], where imaging with surface chemical sensitivity and X-ray photoelectron spectroscopy (XPS) from a 180 nm diameter X-ray spot were performed. A SPEM synchrotron source X-ray beam was focused at the sample down to a 180 nm spot using Fresnel zone plate optics. Samples could be raster scanned with respect to the microprobe to produce chemical maps of specific elements, or to acquire XPS spectra from specific points on the sample surface. Photoelectrons were collected with a SPECS-PHOIBOS 100 hemispherical analyzer, and detected by a 48-chanel electron detector. Photon energy of 1072.3 eV was used for these measurements.

The characterization of nanocomposites was performed by Fourier-transform infrared spectroscopy (FTIR) using a JASCO 6100 FTIR spectrometer (Tokyo, Japan). FTIR spectra were recorded in the spectral range of 4000–400 cm^−1^, with a resolution of 4 cm^−1^, using the KBr pellet technique. The collected spectra were analyzed with Jasco Spectra Manager v.2 software, a soft Spectra Manager Version 2.05.03, copyright 2002-2006, Jasco Corporation.

Total surface area (*S_t_*) and pore radius (*R_m_*) of the samples were obtained from N_2_ adsorption–desorption isotherms (measured at −196 °C), using the BET method for *S_t_*, and Dollimore-Heal model for porosity parameters. The isotherms were recorded by a Sorptomatic 1990 apparatus (Thermo Electron Corporation, Waltham, MA, USA).

Raman spectra were obtaining by using a confocal Raman microscope Invia (Renishaw, UK), endowed with a 533 nm laser, a RenCam CCD detector, 1024 × 256 pixels (200–1060 nm), and an encoded xyz stage (replacement precision: 100 nm), using an 1800 L/mm grating. The spectra were analyzed using the Wire^®^ 4.0 software, which was affiliated with the Raman spectrometer, to elaborate the curve fit.

SEM measurements were carried out by a scanning electron microscope Vega II (Tescan, Czech Republic) at 20 KV of voltage acceleration. It was equipped with Bruker microanalysis and QUANTAX 400 software of analysis, as well as a detector STEM for the acquisition of images in brightfield and in darkfield.

### 2.4. Analysis of Adsorption Process

The adsorption process was performed under static conditions by providing a contact of a synthetic aqueous solution of tartrazine with the synthesized nanocomposite. The experiment was carried out in a Berzelius beaker. The mixture was stirred at 400 rpm/min for a certain period of time, after which the two phases were separated by a magnet. The solute analysis was conducted using the PG Instruments T80 UV-VIS spectrophotometer (Leicestershire, UK), reading the absorbance at 443 nm.

The efficiency of the adsorption process could be determined from the relation:

(1) $$\eta \left(\%\right)=\frac{\left(C_{0}-C_{t}\right)}{C_{0}}100$$

where: *η* (%) represents the degree of tartrazine removal, *C*_0_ and *C_t_* (mg L^−1^) represent the concentrations of the pollutant in the solution at the initial moment and at time *t* (min).

### 2.5. Photocatalytic Degradation of Tartrazine

#### 2.5.1. Tartrazine Photodegradation

The photodegradation of tartrazine was investigated under simulated sunlight irradiation using an Osram Ultra Vitalux 300WE27 (Osram Ultra Vitalux 300 W E27, Munich, Germany). The lamp was placed 20 cm away from a 1 cm cell to irradiate the sample from the top. The radiant flux (40 mW/cm^2^) was measured at the surface of the sample being treated with a radiometer (Cole-Parmer Instrument Co.; model 9811-50, Vernon Hills, IL, USA).

The sample was illuminated by the lamp during different periods of time: 1, 2, 5, 8, 12 and 20 min, respectively. To keep the same temperature conditions, the experiment for absorption was performed simultaneously, but the cell was covered with Aluminum foil to avoid lamp irradiation.

#### 2.5.2. Determination of Photodegradation Efficiency

UV-Vis spectrophotometry (UV-Vis)

The absorbance spectra of the investigated samples were recorded on a dual beam UV-Vis spectrophotometer (Perkin Elmer, model Lambda 650, Waltham, MA, USA) in a 10 mm optical path quartz cuvette (1 mL) (Hellma, model 100-QS, Müllheim, Germany). The spectra were collected over the wavelength range between 300 nm and 800 nm.

The total volume of all examined solutions was 3 mL and contained 5 mg L^−1^ or 10 mg L^−1^ of tartrazine in the case of MWCNT-COOH/Fe_3_O_4_ or that of MWCNT-COOH/Fe_3_O_4_/NiO, respectively, as well as a proper amount of CNTs that were filtrated from the solution before the measurements by the use of a paper filter.

Thermal lens spectrometry (TLS)

**Figure 1:**
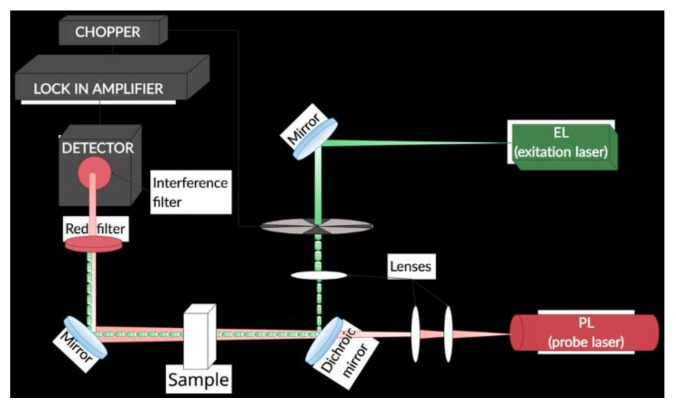
TLS experimental setup used in the study.

The measurements were performed by a homemade dual-beam TLS spectrometer (TLS) (Figure 1).

A diode-pumped solid-state laser (ACX-HTSK, OXXIUS S.A., France) was used as the excitation beam source that operated at 405 nm wavelength and provided 5 mW output power at the position of a 10 mm rectangular quartz detection cell (Starna Scientific Ltd., model 1/Q/10, Pfungstadt, Germany). The induced thermal lens was probed by a He-Ne laser (UNIPHASE, Model 1103P, Edmund Optics, Barrington, NJ, USA) of 2 mW output power at 632.8 nm wavelength. An amplified photodiode (PDA 36A-EC, THORLABS, Newton NJ, USA), placed behind a pinhole and an interference filter 632.8 nm, (FL632.8-1 MELLES GRIOT, USA), monitored the probe beam intensity changes. The photodiode was connected to a lock-in amplifier (STANFORD RESEARCH INSTRUMENTS, SR830 DSP, Sunnyvale, CA, USA). The excitation beam was modulated by a mechanical chopper (SCIENTIC INSTRUMENTS, Control unit model 300C, chopping head model 300CD, chopping disks model 300H, Trowbridge Wiltshire, UK) at 7 Hz, which provided the maximum signal to noise ratio. The excitation beam was focused by a 25 mm diameter lens of 100 mm focal distance (VIS 0° Coated Double-Convex (DCX) Lenses, EDMUND OPTICS, USA) and directed onto the dichroic mirror (HR488+514, HT633/45°, LASER COMPONENTS) by a set of broadband, flat mirrors (400–750 nm, BB1-E02 THORLABS). The probe beam was collimated in a diameter of 3 mm using two lenses (VIS 0° Coated Double-Convex (DCX) Lenses, EDMUND OPTICS, Barrington, NJ, USA) of 30 and 100 mm, respectively. The dichroic mirror enabled overlapping and collinear propagation of the excitation and probe beams and directed them onto the detection cell, before reaching the photodiode.

Each measurement was repeated five times and the average value of the signal, as well as its standard deviations, were calculated.

## 3. Results

### 3.1. Characterization of Synthesized Nanocomposite

#### 3.1.1. XRD Analysis

**Figure 2:**
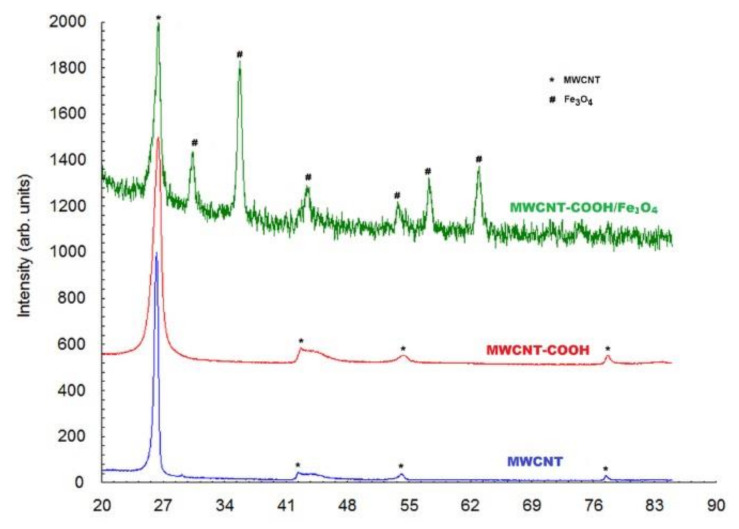
XRD of MWCNT, MWCNT-COOH and MWCNT-COOH/Fe_3_O_4_.

Figure 2 presents the X-ray patterns of diffraction for MWCNT and MWCNT-COOH nanocomposites. Although they are similar in shape, it can be noticed that the breadth of the diffraction lines for MWCNT-COOH was larger. From the Full Width at Half Maximum (FWHM) of the diffraction peaks, the crystallite size was evaluated using the Scherrer relation. The following values were obtained: D = 169 A for MWCNT and 84 A for MWCNT-COOH, respectively. The diffraction peak chosen for determining the crystallites size was 26.4°, which corresponded to the crystallographic plane (0 0 2). This meant that the size of the crystallites represented the length of the MWCNT.

On the basis of the X-ray pattern diffraction of CNT-COOH/Fe_3_O_4_ it was observed that both MWCNT and Fe_3_O_4_ were present in the bio-composite. In the case of MWCNT, only the most intense diffraction peak of 26.4° was observed. The other small diffraction lines were blurred by the corresponding diffraction peaks of Fe_3_O_4_. The corresponding diffraction peaks of Fe_3_O_4_ were quite well highlighted and the crystallite size of Fe_3_O_4_ was found to be 194 Å.

**Figure 3:**
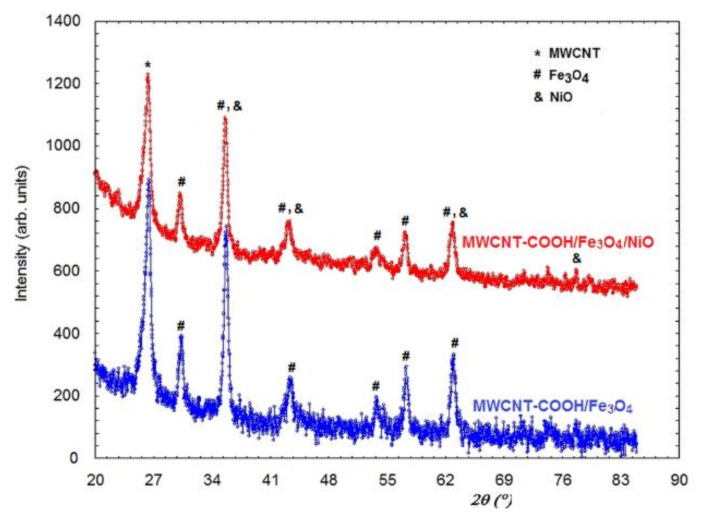
XRD of MWCNT-COOH/Fe_3_O_4_ and MWCNT-COOH/Fe_3_O_4_/NiO.

Figure 3 presents the X-ray diffraction patterns for MWCNT-COOH/Fe_3_O_4_ and MWCNT-COOH/Fe_3_O_4_/NiO. It can be seen that the two diffractograms are very similar. The diffraction maxima for NiO highlighted by SPEM did not appear explicitly in the X-ray powder diffraction pattern because the most intense diffraction lines of NiO partially overlapped the diffraction peaks of Fe_3_O_4_ (Figure 3). The crystallite size of this sample was 161 Å. It was also possible that Ni occurred in the sample, but could be very scattered and, thus, there was not clear evidence of NiO diffraction lines.

#### 3.1.2. SPEM Analysis

**Figure 4:**
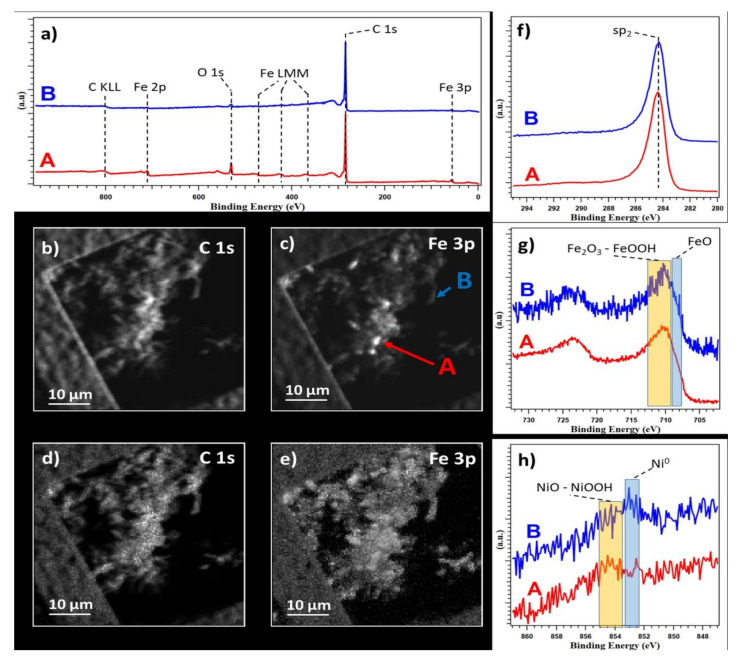
(**a**) Survey spectra. (**b**,**c**) Photoelectron maps acquired at C 1s and Fe 3p edges, respectively, on the top and left sides of the images the TEM grid used to hold the sample is visible. (**d**,**e**) Chemical distribution maps for C 1s and Fe 3p extracted from (**b**,**c**,**f**–**h**) C 1s, Fe 2p, and Ni 2p XPS spectra. All spectra are normalized and were acquired in point A and B highlighted in (**b**).

The measurements were performed on MWCNT-COOH/Fe_3_O_4_/NiO. To avoid modifications in the chemistry, the sample was analyzed without any cleaning procedure in a vacuum. Figure 4a shows the survey spectra acquired on different points. Only C, Fe, and O were visible on the nanotubes bundle. Figure 4b,c show the same area of the sample mapped at different core levels, C 1s and Fe 3p, respectively. The presented maps contain both chemical distribution, and topographic information. In the Fe 3p map, small regions, a few microns wide, showed a higher intensity compared to the same regions in the C 1s. These indicated lack of uniform distribution of Fe and the presence of regions with higher iron concentration. By removing the topographic information (see reference for more detail on the procedure [37], maps (d) and (e) are obtained from C 1s and Fe 3p maps, respectively. In the Figure 4d it was possible to observe a uniform carbon distribution, while the Fe 3p maps confirmed the presence of an area with more Iron.

XPS spectra were acquired on two points, marked as A and B in Figure 4c, and corresponding to high and low iron concentrations, respectively. C 1s spectra, Figure 4a,f, were identical on both points, indicating a uniform C chemistry in the sample, confirming the uniform C distribution highlighted by the C 1s map, and showing a clear *sp2* peak at 283.3 eV from CNT. In the literature, a tail at higher binding energy is associated to C bonded to O as with adsorbed CO_2_ typical of samples not cleaned in a vacuum, and this was also visible [38,39].

The Fe 2p spectra showed identical chemical composition in both points, as can be seen in Figure 4g, except for the signal intensity, confirming the difference in concentration highlighted by the map and survey. A clear shoulder in the spectra around 708–709 eV indicated the presence of FeO [40], while the main components were centered around 709–712 eV, and compatible with both Fe_2_O_3_ and FeO, or a mixture of the two [40].

On both points, together with C, and Fe, a small trace of Ni was also barely visible, Figure 4h, but not visible in the survey (Figure 4a) and in a corresponding Ni map (not showed). Here the spectra acquired showed a difference in chemistry from point to point. In both points, a component compatible with both NiO and/or NiOOH around 853.5–855 eV was present, but in point B a second component at 852.5–853.5, compatible with Ni metallic, was clearly more pronounced than in point A [40].

#### 3.1.3. FTIR Analysis

**Figure 5:**
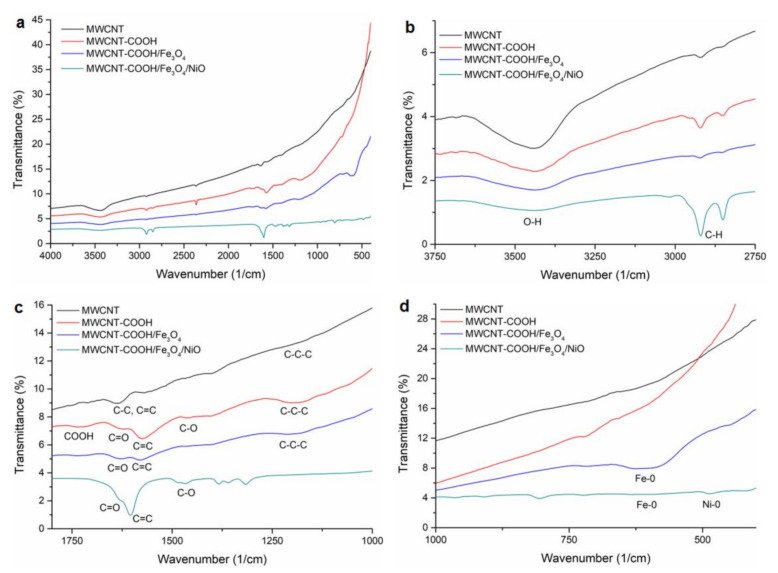
The FTIR spectra of MWCNT, MWCNT-COOH, MWCNT-COOH/Fe_3_O_4_ and MWCNT-COOH/Fe_3_O_4_/NiO: (**a**) 4000–400 cm^−1^ spectral domain; (**b**) 3750–2750 cm^−1^ spectral domain; (**c**) 1800–1000 cm^−1^ spectral domain; (**d**) 1000–400 cm^−1^ spectral domain.

FT-IR measurements were performed to identify the presence of functional groups on the MWCNT, oxidized MWCNT, the MWCNT–COOH/Fe_3_O_4_ and the MWCNT–COOH/Fe_3_O_4_/NiO surfaces. The obtained spectra are presented in Figure 5.

In the spectrum of MWCNT, the peaks that appeared at 3430, 1623 and at 1398 cm^−1^ were assigned to stretching vibrations of O–H groups from the surface and from adsorbed water [41], the vibrations at 2907 and 2846 cm^−1^ were attributed to C-H groups and the peak at 1556 cm^−1^ revealed the stretching vibration of C=C. These bands were observed in all spectra.

The characteristic vibrational band of polar functional –COOH groups generated after chemical oxidation of MWCNT appeared in the FT-IR spectrum of MWCNT-COOH at 1690 cm^−1^. At 1582 cm^−1^ the stretching vibration of the H-bonded C=O group, at 1528 and 1162 cm^−1^ stretching vibration of C=C and C-C-C bonds from carbon nanotube structure appeared [42,43].

These bands were shifted in the MWCNT–COOH/Fe_3_O_4_ spectrum at 1725, 1613, 1567 and 1201 cm^−1^, respectively, probably due to the interactions of magnetic nanoparticles on the MWCNT-COOH surface. The characteristic absorption band for Fe-O bond from Fe_3_O_4_ situated in the spectral range 375–650 cm^−1^ was observed at 596 cm^−1^ and revealed the presence of magnetite nanoparticles on the surface of oxidized MWCNTs [44].

On the spectrum of MWCNT–COOH/Fe_3_O_4_/NiO the spectral bands at 3424 cm^−1^ with low intensity, and at 2920 and 2850 cm^−1^, corresponding to stretching vibrations of O–H and to C-H groups, respectively, could be observed. The spectral bands attributed to stretching vibration of H-bonded C=O group and to stretching vibration of C=C and C-C-C bonds from carbon nanotube structure appeared, slightly shifted, at 1690 as a shoulder, 1630, 1603, and the band from 1162 cm^−1^ disappeared. The absorption band of the Fe-O bond appeared, with very low intensity, at 588 cm^−1^. The Ni-O vibration appeared at 480 cm^−1^ [45].

#### 3.1.4. Raman Analysis

Raman spectroscopy is an important non-destructive technique used to characterize the microstructure of carbonaceous materials.

**Figure 6:**
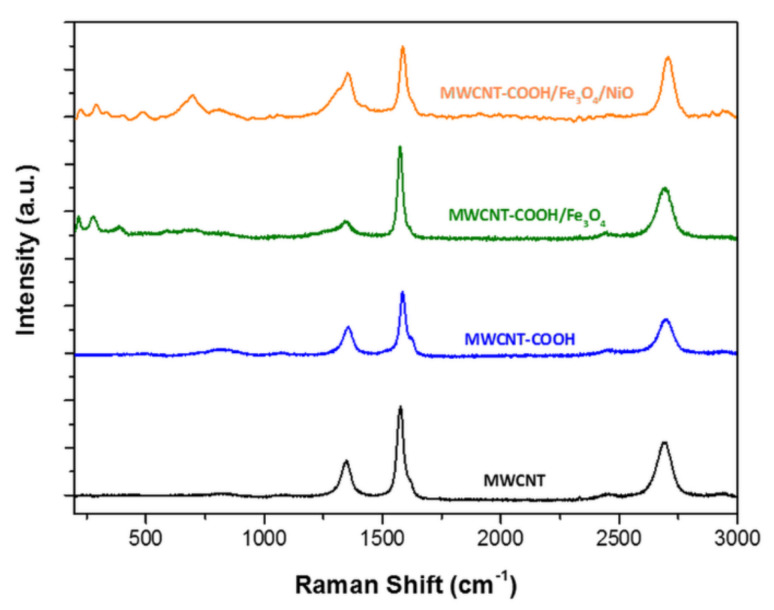
Raman spectra, using a 1800 lmm^−1^ grating, in the 200–3000 cm^−1^ spectral range of MWCNT (black line), MWCNT–COOH (blue line), MWCNT–COOH/Fe_3_O_4_ (green line) and MWCNT–COOH/Fe_3_O_4_/NiO (orange line).

The spectra of all the samples showed two prominent peaks at about 1350 cm^−1^ and 1576 cm^−1^, corresponding to the D and G bands of graphite, respectively, and there was a shoulder at G band near 1620 cm^−1^ (D’ band) for MWCNT-COOH, MWCNT–COOH/Fe_3_O_4_ and MWCNT–COOH/Fe_3_O_4_/NiO samples, as reported in Figure 6.

The D band is related to defective CNT and non-crystalline carbon (sp^3^ diamond-like carbons) and it is associated with disordered carbon (sp^3^ hybridized carbon) that does not have in-plane symmetry with the graphene, whereas the G band corresponds to the well-ordered sp^2^ carbon atoms of graphitic materials.

The ratio between the intensities of D band and G band (I_D_/I_G_) is commonly used to evaluate the disorder degree of graphitic materials (average size of C–C sp^2^ domains). In MWCNT pristine (black line) the I_D_/I_G_ ratio was around 0.38 and in MWCNT-COOH (blue line) it was around 0.42. An increase of I_D_/I_G_ suggested a loss of aromaticity (oxidation of sp^2^ carbon hybridization) in the rings of MWCNTs. The loss of aromaticity could be caused by defects induced by the change in the hybridization of C–C sp^2^ for C–C sp^3^ due to the presence of oxygen. The spectrum of the MWCNT–COOH/Fe_3_O_4_ sample (green line) exhibited some extra peaks at lower wavenumbers 217, 278, 387, 589, 679 cm^−1^, which were consistent with the vibration modes of Fe–O bonds in Fe_3_O_4_ and Fe–C bonds between Fe_3_O_4_ and MWCNT. The Raman spectrum of MWCNT–COOH/Fe_3_O_4_/NiO (orange line) showed new peaks at lower wavenumbers 226, 291, 484, 698, 809 and 1056 cm^−1^ that could be attributed to the vibration modes of Fe_3_O_4_ and NiO. The I_D_/I_G_ ratio of around 0.62 indicated an increase of the number of defects (disordered carbon) in the tube walls, due to the presence of NiO and Fe_3_O_4_ in the nanocomposite [46].

#### 3.1.5. Surface Area and Porosity Analysis

**Figure 7:**
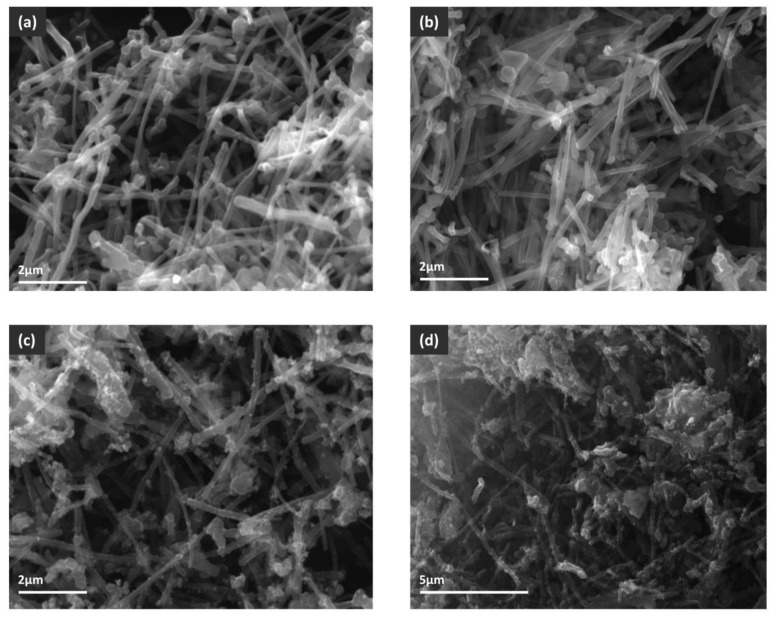
SEM images of (**a**) MWCNT, (**b**) MWCNT-COOH, (**c**) MWCNT-COOH/Fe_3_O_4_ and (**d**) MWCNT-COOH/Fe_3_O_4_/NiO.

The total surface areas of MWCNT and MWCNT-COOH were similar, with a slight increase for carboxylated nanotubes (18 m^2^ g^−1^ compared to 15 m^2^ g^−1^), suggesting that the surface oxidation did not change the morphologies of the MWCNT samples. This observation was also sustained by the SEM images (Figure 7). For MWCNT-COOH/Fe_3_O_4_ the measured surface area was 41 m^2^ g^−1^. The higher value compared to nanotubes was most probably due to the presence of magnetite, a porous oxide that adds supplementary surface to the composite material. For the CNT–MWCOOH/Fe_3_O_4_/NiO sample the surface area decreased to only 11 m^2^ g^−1^, suggesting that either the nanotubes agglomerated, losing surface, or the oxides’ porous structure somehow collapsed, or the pores of the MWCNT-COOH/Fe_3_O_4_ framework were occupied by less porous NiO nanoparticles, leading, thus, to the decrease of surface area. By analyzing the SEM images, it could be observed that, for MWCNT-COOH/Fe_3_O_4_, the majority of the magnetite was deposited as small grains attached to the nanotubes, bringing additional surface area to the composite. In the case of MWCNT–COOH/Fe_3_O_4_/NiO big particles, or agglomerations of oxide grains, could be seen, clogging the interspace between the nanotubes, and most probably leading to the observed decrease of surface area. Taking into account that the MWCNT–COOH/Fe_3_O_4_/NiO composite was prepared from MWCNT–COOH/Fe_3_O_4_ by additional deposition of NiO, it could be concluded that, during preparation, the nickel oxide did not disperse uniformly on the surface, so that the resulting composite was, thus, less porous than the starting one. Regarding the pore size distribution, a very large and similar distribution was observed for all samples, the pore size being situated in the 4–28 nm domains. MWCNT and MWCNT-COOH are not porous materials in the classical acceptation of this term. In their cases, the porosity is formed between the intertangled nanotubes, this being the explanation for the large and non-uniform distribution of pore size. The pore size distribution was not changed by the presence of oxides because, most probably, their pore sizes were in the same range.

#### 3.1.6. Morphological Characterization

SEM images of all analyzed samples are presented in Figure 7.

**Figure 8:**
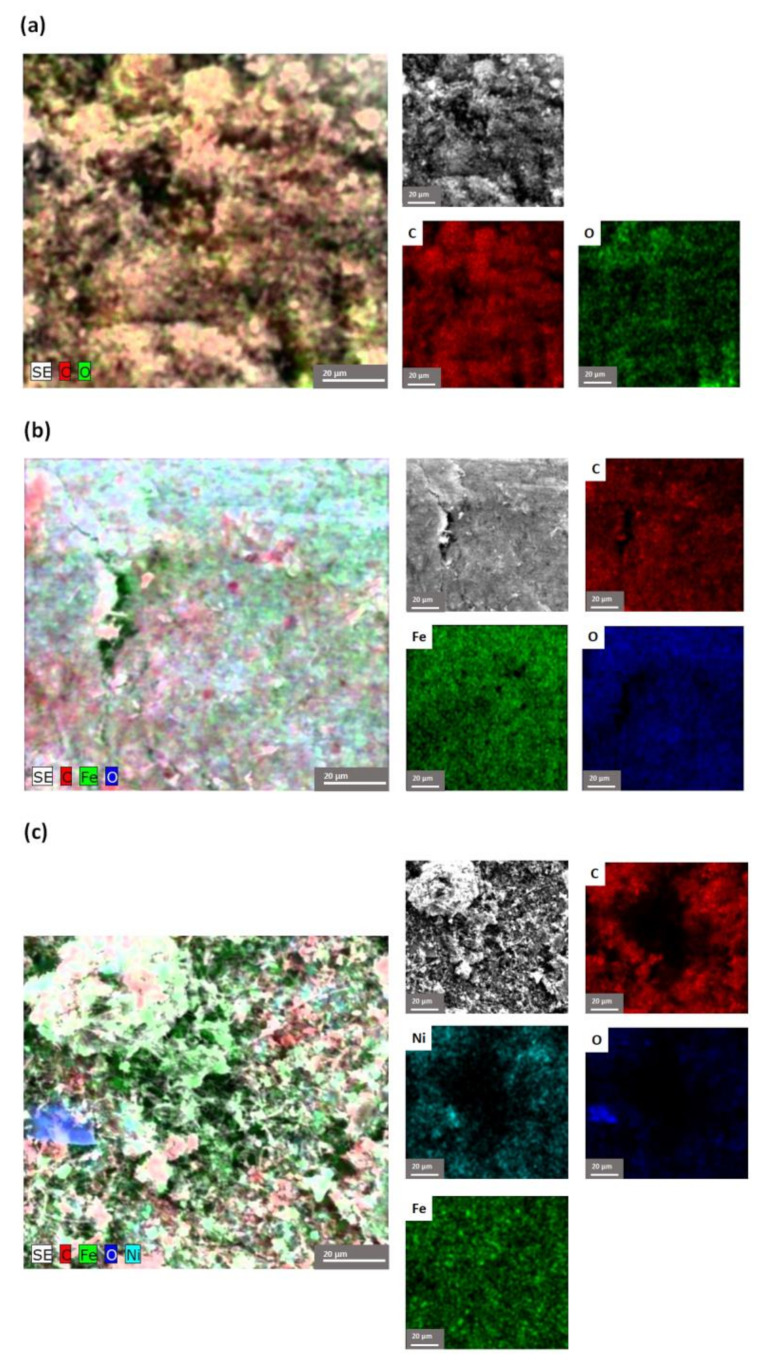
EDX mapping pattern of the element distribution and the quantitative analysis spectra of: (**a**) MWCNT-COOH, (**b**) MWCNT-COOH/Fe_3_O_4_ and (**c**) MWCNT-COOH/Fe_3_O_4_/NiO.

The compositional determination, by EDX, in the marked areas, indicates the presence of the elements belonging to all the component phases of the nanocomposites, as shown in Figure 8. The corresponding EDX spectra of the nanocomposites display the distribution in the chosen sample of the elements and, implicitly, of the component phases. The presence of all the elements of the component phases, both metallic and organic, were found.

### 3.2. Testing of MWCNT-COOH/Fe_3_O_4_ and MWCNT-COOH/Fe_3_O_4_/NiO Nanocomposites for Removal of Tartrazine from Synthetic Aqueous Solutions by Adsorption

In order to establish the optimal retention conditions of tartrazine, the influences of some physico-chemical parameters on the adsorption process were examined. Among them were the initial pHs of the dye solution, the temperature at which the adsorption was performed, adsorbent dose, and contact time, as well as the initial concentration of dye.

#### 3.2.1. The Influence of pH on the Adsorption Process

In order to determine the optimal pH at which tartrazine is adsorbed, 5 mg of adsorbent material was stirred at 400 rpm with 5 mL of tartrazine solution of concentration 30 mg L^−1^ at different pH values (2 and 10) at room temperature (25 °C), for 30 min. The pH of the tartrazine solution was adjusted with 0.1 M HCl or 3 M NaOH. At the end of the adsorption process, the two phases were separated and the solute analyzed.

**Figure 9:**
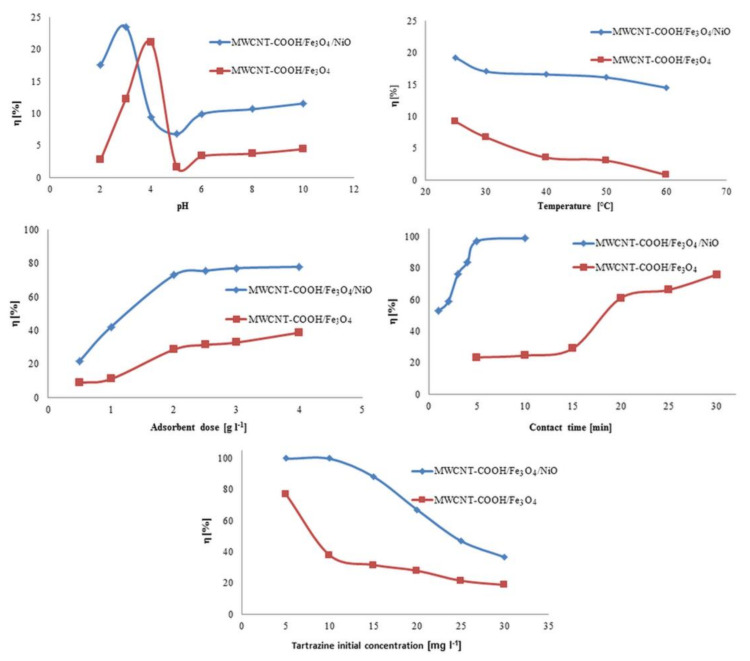
The effect of the experimental parameters on the adsorption degree of tartrazine on studied adsorbents.

The results showed that the degree of tartrazine clearance varied, depending on the pH value of the solution (Figure 9). For the MWCNT-COOH/Fe_3_O_4_/NiO nanocomposite the best degree of tartrazine removal was obtained at pH 3, and for the MWCNT-COOH/Fe_3_O_4_ nanocomposite it was at pH 4. At the optimum pH values, the degree of tartrazine removal from the synthetic aqueous solution was approximately 20% for both adsorbents studied. A similar pH value was obtained for the removal of tartrazine on chitin and chitosan [47], and also on sawdust [48].

#### 3.2.2. The Influence of Temperature on the Adsorption Process

Samples of 5 mg adsorbent were mixed with 5 mL of tartrazine solution of concentration 30 mg L^−1^ and then stirred at 400 rpm for 30 min at temperatures between 25 and 60 °C. The pH of the tartrazine solution was 3 if the adsorbent used to remove tartrazine was MWCNT-COOH/Fe_3_O_4_/NiO and 4 if the adsorbent was MWCNT-COOH/Fe_3_O_4_. The solute was also analyzed separately using an external magnet.

It was observed that the degree of removal of tartrazine decreased with increasing temperature (Figure 9) and, for this reason, for future studies the optimum temperature should be fixed at 25 °C. This could be explained by the fact that temperature increased the bonds between the dye and the active sites on the adsorbent, due to their weakening [48]. Similar results were obtained for the removal of Direct Red 23 and Direct Red 80 dyes by orange peel adsorbent and for removal of tartrazine and sunset yellow onto activated carbon derived from *Cassava sievate* biomass [49,50].

#### 3.2.3. Influence of Adsorbent Dose on the Adsorption Process

The effect of the adsorbent dose on the tartrazine adsorption process was followed by stirring at 400 rpm, at a temperature of 25 °C, for 30 min, 5 mL of 30 mg L^−1^ solution of tartrazine solution with different amounts of adsorbent. The doses of MWCNT-COOH/Fe_3_O_4_ and MWCNT-COOH/Fe_3_O_4_/NiO tested were between 0.5 and 0.4 g L^−1^. The tartrazine solution had a pH of 3 when using MWCNT-COOH/Fe_3_O_4_/NiO as an adsorbent and pH 4 of the MWCNT-COOH/Fe_3_O_4_ nanocomposite.

As the dose of adsorbent increased from 0.5 to 4 g L^−1^, the degree of removal of tartrazine increased on both types of adsorbents (Figure 9). This increase in the degree of removal was due to the availability of several active adsorption sites. For economic reasons, for subsequent studies, 1 g L^−1^ was chosen as the adsorbent dose for both MWCNT-COOH/Fe_3_O_4_ and MWCNT-COOH/Fe_3_O_4_/NiO.

#### 3.2.4. Influence of Initial Tartrazine Concentration on the Adsorption Process

Studies on the influence of the initial concentration of tartrazine on its adsorption on selected adsorbents were performed using 5 mg nanocomposite with 5 mL of tartrazine solution of concentrations between 5 and 30 mg L^−1^, at 25 °C, for 30 min using a magnetic set at 400 rpm.

In Figure 9 it can be seen how the degree of tartrazine clearance decreased with increasing initial dye concentration. In the case of using the MWCNT-COOH/Fe_3_O_4_/NiO nanocomposite as an adsorbent, the optimal initial concentration was 10 mg L^−1^, and in the case of the MWCNT-COOH/Fe_3_O_4_ nanocomposite it was 5 mg L^−1^. These concentrations were used for subsequent studies.

#### 3.2.5. The Influence of Contact Time on the Adsorption Process

The determination of the optimal time required to remove tartrazine from aqueous solutions in the case of the two adsorbents was performed as follows: 5 mL of tartrazine solution of concentration 10 mg L^−1^ at pH 3 were stirred with 5 mg of MWCNT-COOH/Fe_3_O_4_/NiO at 400 rpm, at 25 °C, reaction time between 1 and 10 min and 5 mL of tartrazine solution of concentration 5 mg L^−1^ at pH 4 were stirred with 5 mg of MWCNT-COOH/Fe_3_O_4_ at 400 rpm, at 25 °C, reaction time between 5 and 130 min.

From Figure 9, it can be seen that the removal efficiency of tartrazine depended on the adsorbent used. If the MWCNT-COOH/Fe_3_O_4_/NiO nanocomposite was used as an adsorbent, the degree of removal of tartrazine increased in the first 5 min to 97.3%. When using the MWCNT-COOH/Fe_3_O_4_ nanocomposite as an adsorbent, the degree of tartrazine removal increased with increased contact time after 15 min.

### 3.3. Photocatalytic Performance of Synthesized Nanocomposites

The best conditions obtained for tartrazine’s removal by adsorption were used to test the photocatalytic performance of synthetized nanocomposites.

The amount of tartrazine was calculated using the calibration curve constructed for the concentration range 0–10 mg L^−1^, using the maximum absorption wavelength (428 nm). Thus, the linear regression equation for tartrazine was y = 0.038× (R^2^ = 0.9735) with the detection limit (LOD) at 0.25 mg L^−1^.

**Figure 10:**
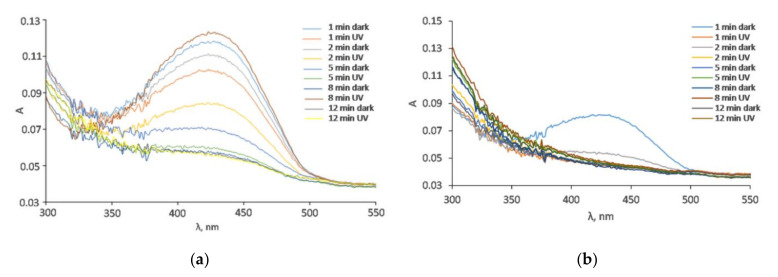
The absorption spectra of tartrazine with the addition of (**a**) MWCNT-COOH/Fe_3_O_4_, (**b**) MWCNT-COOH/Fe_3_O_4_/NiO and illumination with different sunlight irradiation times.

**Figure 11:**
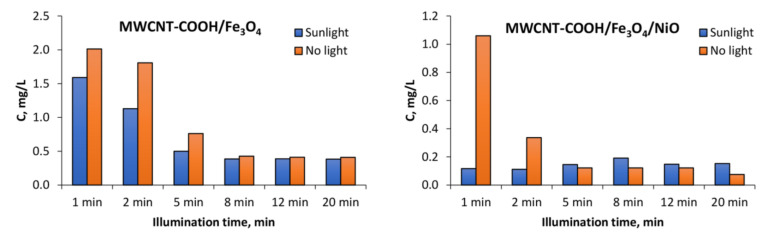
Decrease in tartrazine amount of leftover concentration in the solution determined by UV-vis spectrometry because of degradation by the use of synthesized nanocomposites and simulated sunlight illumination.

**Figure 12:**
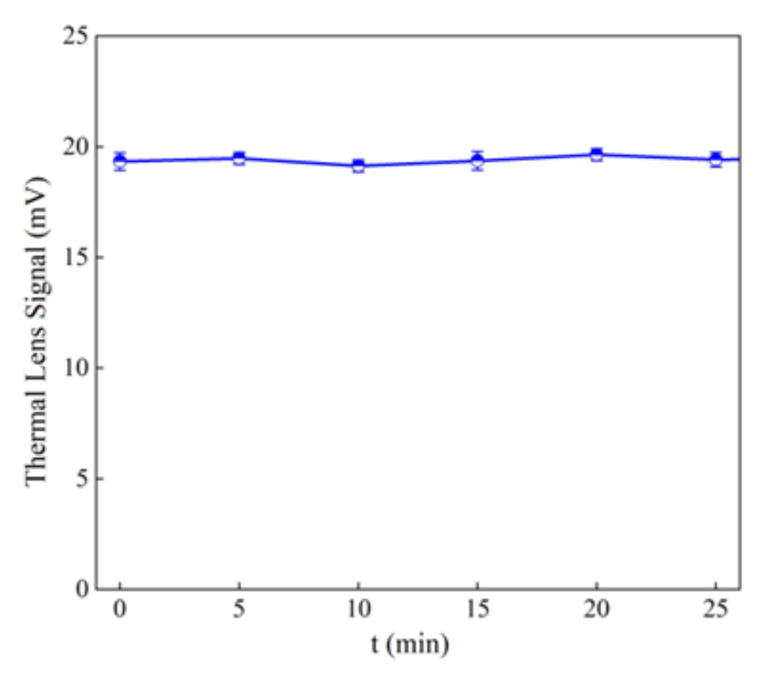
Thermal lens signal of the tartrazine under Uv illumination without any catalyst.

The degradation of 10 mg L^−1^ tartrazine after the addition of 1 g L^−1^ MWCNT-COOH/Fe_3_O_4_ with and without illumination can be observed in Figure 10 and Figure 11. In Figure 12 can be observed that under UV illumination without any catalyst, there was no tartrazine signal modification within of exposure.

In the case of the MWCNT-COOH/Fe_3_O_4_ nanocomposite, it can be seen that the presence of simulated sunlight radiation increased the rate of tartrazine degradation by about 20% in the first 2 min of irradiation, and after 5 min it dropped to 10%. After 8 min of UV irradiation, the degradation process in both cases (with and without UV irradiation) was not effective anymore. The amounts of leftovers were constant with time and were at the level of 150% of the blank value, which indicated that the tartrazine was not totally decomposed.

**Figure 13:**
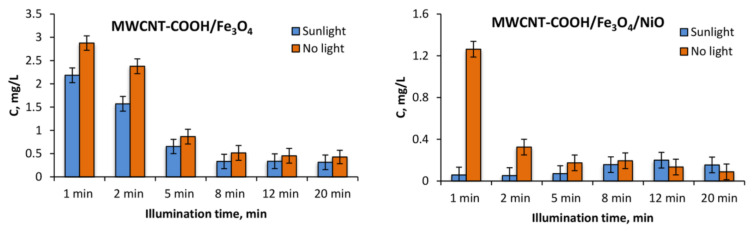
Decrease in tartrazine amount of leftovers concentration in the solution determined by TLS as a result of degradation by the use of synthesized nanocomposites and sunlight illumination.

In the case of using the MWCNT-COOH/Fe_3_O_4_/NiO nanocomposite, it is seen that in the presence of simulated sunlight irradiation the detected signal had already reached the value of blank after 1 min of UV irradiation, whereas in the case without simulated sunlight irradiation such a condition was obtained after 5 min of MWCNT-Fe-Ni interaction. It can be concluded from these results that the high LOD (0.25 mg L^−1^) of the UV-Vis spectrometric technique meant accurate and reliable results at very low dye concentration (after 8 min irradiation) could not be provided. The requirement of accurate measurements at very low concentration of dye could be satisfied by the TLS technique, which is more sensitive than UV-Vis spectrophotometry. For TLS analysis, the calibration curve was constructed for the same concentration range of tartrazine as in the case of UV-Vis analysis. For this technique, the linear regression equation was y = 5.0765× + 1.3058 (R^2^ = 0.9941) with LOD 0.01 mg L^−1^ which was 25 times lower than in the case of UV-vis spectrophotometry, Figure 13.

The results obtained in both cases coincided with those obtained by UV-vis spectrometry. However, because of a lower LOD in case of measurement by TLS, more accurate and reliable results were obtained.

Collectively, our results demonstrated that MWCNT-COOH/Fe_3_O_4_ and MWCNT-COOH/Fe_3_O_4_/NiO nanocomposites exhibited greatly improved photocatalytic activity on the degradation of dyes. The advanced photocatalytic performances were attributed to improved photocurrents and enhanced hole-electron separation rates, due to the combination of MWCNT with metallic oxides [51,52]. This result may be associated with the balance of synergistic effects between MWCNTs and the semiconductor character of Fe_3_O_4_. Grafting functional molecules or groups on the surface of carbon nanotubes is an important way to demonstrate the improvement of their surface characteristics. We can conclude that UV, visible light or sunlight photocatalytic activity of the MWCNT-based nanocomposites is higher than that of the metal oxide or mechanical mixture of the metal oxide and carbon nanotubes [53,54,55]. Under sunlight irradiation electrons and holes are created in the conduction and valence bands, respectively. Subsequently, the free electrons are injected from Fe_3_O_4_ to MWCNTs through the interface between them, leading to a reduction of electron-hole recombination process. Therefore, more carriers can participate in the photocatalytic process.

Although the surface area of MWCNT-COOH/Fe_3_O_4_/NiO was relatively lower than that of MWCNT-COOH/Fe_3_O_4_, a higher photocatalytic activity was demonstrated. This can be justified, because the combination of NiO and MWCNT has oxygen-rich functional groups (e.g., hydroxyl and carboxyl), which can improve the oxidation process and, therefore, the photocatalytic activity [56].

## 4. Conclusions

Absorption and photocatalytic mechanisms of tartrazine degradation were qualitatively distinguished using MWCNT-COOH/Fe_3_O_4_ and MWCNT-COOH/Fe_3_O_4_/NiO nanocomposites as catalysts. In the case of tartrazine removal by adsorption, several parameters that influence the process were investigated, among which pH, temperature, adsorbent dose, initial concentration of the dye solution and contact time were considered. Of the two adsorbents tested, the best degree of removal of tartrazine was obtained with MWCNT-COOH/Fe_3_O_4_/NiO. Alternatively, the TLS technique provided similar results compared to UV-Vis spectrophotometry. However, the lower LOD of the TLS enabled measurements to provide more reliable and accurate results when studying the photodegradation mechanism at low dye concentrations. The enhanced photocatalytic and adsorption performances of MWCNT-COOH/Fe_3_O_4_ and MWCNT-COOH/Fe_3_O_4_/NiO make them promising photocatalysts and adsorbents for wastewater treatment.

## Data Availability

The data presented in this study are available on request from the corresponding author.
